# Novel Antibacterial Materials and Coatings—A Perspective by the Editors

**DOI:** 10.3390/ma16186302

**Published:** 2023-09-20

**Authors:** Hanif Haidari, Krasimir Vasilev

**Affiliations:** 1 Future Industries Institute, University of South Australia, Mawson Lakes, SA 5095, Australia; 2 Biomedical Nanoengineering Laboratory, College of Medicine and Public Health, Flinders University, Bedford Park, SA 5042, Australia

## 1. Introduction

The fight between humans and bacteria has escalated to a new level. The past several decades witnessed an alarming increase in the occurrence of bacterial infections on a global scale, presenting enormous clinical challenges with great morbidity and mortality, as well as financial burden [1]. A major reason for this is the increased prevalence of antibiotic-resistant bacteria and the decline in the development of new antimicrobial agents. These factors have made it challenging to provide effective treatment options for even common bacterial infections [2]. Antibiotics have been crucial in modern medicine for a long time; however, the loss of their effectiveness has jeopardized the fight against infections, particularly when treating wounds, catheters, and implants, which often turn simple infections into life-threatening medical conditions [3,4]. Like never before, there is a pressing demand to act collaboratively and implement a holistic approach to mitigate the ever-growing threat of antimicrobial resistance (AMR) worldwide [5].

This Special Issue focuses on a collection of primary research and review articles in the area of emerging antimicrobial materials for various fields of application, including medicine, agriculture, marine fouling, and food production. This Special Issue is highly timely as it is essential to encourage a collaborative research approach to develop novel antibacterial materials with translational potential and capacity for reaching healthcare providers to assist them in managing bacterial-associated infections. Collectively, over recent years, the scientific and medical communities have made substantial progress in devising new and alternative approaches to combat the threat of superbugs [6]. The focus has been concentrated on non-traditional approaches, primarily because pathogenic bacterial infections are complex phenomena requiring materials with new modes of action with higher potency and strategies to deliver active molecules efficiently to the bacterial targets [7]. So far, progress has been reflected in the utilization of nanotechnology to design and develop antibacterial nanomaterials and surface-functionalized coatings with novel properties to break infection cycles, either preventing colonization or dispersing established biofilms.

Nanotechnology-based materials have transformed human medicine and health care, especially in infectious disease diagnostics and drug delivery. A variety of materials have been explored, including polymeric nanoparticles, inorganic nanoparticles (silver, copper, and gold), and nanoparticles assisted with stimuli (heat, photon) [7,8]. Traditionally, the mechanism of action of antimicrobial materials has been consistent, either causing contact killing or releasing the biocides when in the vicinity of the device [9]. Current developments in antibacterial materials have utilized newly devised strategies to provide targeted delivery to the infectious environment with high specificity and reduced drug exposure to un-intended sites. Furthermore, increased permeability and transportation to complex infections, including biofilm matrixes, have improved the bioavailability of drugs and multifaceted mechanisms of action, making them more potent and making it more difficult for bacteria to develop resistances. All these mechanisms have been pivotal in decreasing bacterial resistance while improving therapeutic outcomes.

Amongst all of these breakthroughs, silver nanoparticles (AgNPs) have gained tremendous popularity in commercial products and applied research due to their potential to address the challenge of multidrug-resistant infections [10,11]. Silver has broad-spectrum antimicrobial activity that is effective against bacteria, viruses, and fungi. The antibacterial potential of AgNPs has been widely explored and believed to be influenced by nanoparticles’ physiochemical properties such as stability, size, shape, and surface chemistry, as well as release mechanisms. However, the safe delivery of AgNPs has been the main drawback towards clinical translation, with high unwanted silver toxicity, poor stability, and lack of inconsistent antibacterial efficacy being encountered often. Research in this space has been dominated by developing alternative delivery systems. For example, Haidari and colleagues have carried out extensive fundamental and applied studies of AgNP development for targeted and smart delivery systems for wound disinfection [12]. The team has developed ultrasmall AgNPs loaded with a biocompatible stimuli-responsive hydrogel for targeted and enhanced silver delivery to facilitate better bacterial biofilm penetration and dispersion [13,14]. Furthermore, the use of smart hydrogel AgNPs has allowed for the on-demand release of the therapeutics, enabling effective infection controls supported with desirable wound healing outcomes [15]. These studies demonstrate that AgNPs could be used for clinical wound infections, offering a controlled and safe release mechanism that is effective in clearing infection and promoting the recovery of infected wounds without developing bacterial resistance [16,17]. The delivery of AgNPs has been a huge research focus utilizing different kinds of platforms, including pH- and temperature-responsive hydrogels [18], mesoporous silica [19], microneedles [20], and 3D printings [21], all of which show exceptional potential to eliminate infection safely. Other attractive approaches include photothermal therapy of bacteria using near-infrared light (NIR), generating a cocktail of reactive oxygen species and spatial heat and providing a convenient method for ablating bacterial infection with an on-demand release strategy [22,23]. For example, Min and colleagues developed a dual-function pH-sensitive hydrogel capable of real-time wound monitoring with on-demand photothermal therapy that resulted in 99% bacterial inhibition, offering a highly innovative platform for smart infection management [24]. This type of approach with real-time sensing and on-demand release is gaining research interest and no doubt will have a significant impact on treating either superficial or deep wound infections.

Another attractive and emerging approach for infection control is the utilization of liquid metals such as Galium. Progress in this field was recently summarised and critically discussed in an excellent review published in ACS Nano [25]. The liquid metals can be easily deposited as coatings via established conventional techniques to be used as a suspension. They can also be combined with other metals to create fascinating shape-changing nanoparticles capable of destroying established biofilms [26,27]. We recommend this space be monitored since the potential of these new materials in fighting resistant pathogens is high.

Research in the field continues to evolve, with the discovery and development of new strategies to fight against bacteria occurring regularly. For instance, it is well-known that a large proportion of nosocomial infections are closely associated with medical devices such as ventilators, catheters, injectors, and implantable scaffold devices [28,29]; indeed, infection is one of the main causes of implant failure. For this purpose, a huge variety of antibacterial coatings with different compositions and fabrication strategies have emerged to either prevent or eliminate bacterial attachments. Medical device infections pose a high risk to the patient as they are often undiagnosed at early stages, presenting recurrent and complex infections. As a result, researchers around the world have focused on the development of antibacterial coatings on the surface of medical devices to confer bacterial attachment and colonization [30,31]. It has been shown that this strategy is an effective way to reduce the occurrence of healthcare device-associated infections. This is achieved by modifying the interfacial properties of medical devices without disrupting the bulk properties of materials. Over the past decade, a variety of surface modification or treatment techniques has emerged for the fabrication of antibacterial surfaces, either presenting bacteria-repellent properties [32], contact-killing mechanisms [33], or the release of antimicrobial agents [34]. Antibacterial surfaces have been functionalized by plasma polymerization to improve the functionality of the surface, enabling the binding of antimicrobial agents, which has received considerable research interest and has broad applications.

One effective strategy to employ a robust and clinically acceptable coating is grafting with antimicrobial agents. Release-based antibacterial surfaces are loaded with leachable bactericidal compounds such as antibiotics [35], cationic polymers [36], antimicrobial peptides (AMPs) [37], nitric oxide (NO) [38], and metallic nanoparticles [39]. Avoidance of antibiotic coatings is always encouraged to prevent the issue of bacterial resistance. Taheri et al. developed silver-nanoparticle-immobilized surfaces for the prevention and eradication of pathogenic bacteria, resulting in a promising platform to eliminate clinical signs of infections [40]. Another strategy employed by Bright and colleagues has shown the generation of a sharp nanostructured surface with titanium that effectively ruptures and eliminates pathogens, offering a potential strategy to be employed in medical devices that could help clinicians prevent implant-associated infections and recurrence. It should be noted that recent studies from Vasilev and co-workers have demonstrated that these nanostructures can work synergistically with conventional antibiotics currently used in clinical practice [41]. This discovery can have a tremendous impact on how infections are treated and prevented in the near future. Other strategies employed use a bacteria-repelling mechanism where the surface is functionalized using polymers such as polyethylene glycol and polyoxazoline [42] that have anti-fouling properties or by using zwitterionic polymers such as quaternary ammonium [43]. These approaches are considered biologically safe and effective for biomedical devices. Research in this space continues to adapt and evolve, as does the pursuit of promising antibiotic-free approaches to efficiently target bacteria with high clinical prospects [44,45].

## 2. Conclusions

This extended editorial perspective highlights recent progress and provides a future outlook for novel and emerging antibacterial materials. We have stressed the great importance of developing innovative antibacterial materials and surface coatings that could provide a much-needed solution for clinical infections. Despite the rapid progress in the field, clinical translation of these products is far from sufficient, and there are still many challenges, including clinical efficacy, safety, and regulatory burden. However, taking these discoveries into the next stage requires global interdisciplinary cooperation between researchers, clinicians, and industries. This Special Issue is timely, targeted, and focused, and we hope to encourage and promote imaginative and disruptive strategies to fight against AMR, which is expected to have huge consequences if there are no immediate collective actions.
